# Angiotensin-Converting Enzyme (ACE) level, but not *ACE* gene polymorphism, is associated with prognosis of COVID-19 infection: Implications for diabetes and hypertension

**DOI:** 10.1371/journal.pone.0288338

**Published:** 2023-07-11

**Authors:** Onur Elbasan, Feyza Bayram, Ceyda Dinçer Yazan, Tuğçe Apaydın, Saida Dashdamirova, Hamza Polat, Ebru Arslan, İpek Yılmaz, Nastaran Karimi, Buket Ertürk Şengel, Sultan Seval Yılmaz, Ömer Faruk Çelik, Pınar Ata, Goncagül Haklar, Hülya Gözü

**Affiliations:** 1 Department of Endocrinology and Metabolism, Marmara University Faculty of Medicine, Istanbul, Turkey; 2 Department of Medical Genetics, Marmara University Faculty of Medicine, Istanbul, Turkey; 3 Department of Infectious Diseases and Microbiology, Marmara University Faculty of Medicine, Istanbul, Turkey; 4 Department of Biochemistry, Marmara University Faculty of Medicine, Istanbul, Turkey; Western Norway University of Applied Sciences: Hogskulen pa Vestlandet, NORWAY

## Abstract

**Background:**

The renin-angiotensin-aldosterone system was shown to be activated in severe COVID-19 infection. We aimed to investigate the relationship between angiotensin converting enzyme (ACE) levels, *ACE* gene polymorphism, type 2 diabetes (T2DM), and hypertension (HT) and the prognosis of COVID-19 infection.

**Methods:**

This cross-sectional study analyzed the clinical features of adult patients with SARS-CoV-2 infection. *ACE* gene analysis and ACE level measurements were performed. The patients were grouped according to *ACE* gene polymorphism (DD, ID or II), disease severity (mild, moderate, or severe), and the use of dipeptidyl peptidase-4 enzyme inhibitor (DPP4i), ACE-inhibitor (ACEi) or angiotensin receptor blocker (ARB). Intensive care unit (ICU) admissions and mortality were also recorded.

**Results:**

A total of 266 patients were enrolled. Gene analysis detected DD polymorphism in the *ACE 1* gene in 32.7% (n = 87), ID in 51.5% (n = 137), and II in 15.8% (n = 42) of the patients. *ACE* gene polymorphisms were not associated with disease severity, ICU admission, or mortality. ACE levels were higher in patients who died (p = 0.004) or were admitted to the ICU (p<0.001) and in those with severe disease compared to cases with mild (p = 0.023) or moderate (p<0.001) disease. HT, T2DM, and ACEi/ARB or DPP4i use were not associated with mortality or ICU admission. ACE levels were similar in patients with or without HT (p = 0.374) and with HT using or not using ACEi/ARB (p = 0.999). They were also similar in patients with and without T2DM (p = 0.062) and in those with and without DPP4i treatment (p = 0.427). ACE level was a weak predictor of mortality but an important predictor of ICU admission. It predicted ICU admission in total (cutoff value >37.092 ng/mL, AUC: 0.775, p<0.001).

**Conclusion:**

Our findings suggest that higher ACE levels, but not *ACE* gene polymorphism, ACEi/ARB or DPP4i use, were associated with the prognosis of COVID-19 infection. The presence of HT and T2DM and ACEi/ARB or DPP4i use were not associated with mortality or ICU admission.

## Introduction

The renin-angiotensin-aldosterone system (RAAS) is considered to have an important role in the regulation of blood pressure in which the ACE2 and ACE/Angiotensin (Ang) 2 pathways should be balanced. Elevated levels of Ang 2 occurring due to RAAS activation by binding to AT1R (Angiotensin 2 type 1 receptor) may result in tissue damage, inflammation, fibrosis, edema, thrombosis, hypertension and atherosclerosis [1]. On the other side of the scale, ACE2 is responsible for the cleavage of Ang 1 to Ang 1–9 and Ang 2 to Ang 1–7 peptides; thus, blood pressure is normalized [2, 3]. AT1R, which is the target of Ang 2, coexists to form a cell membrane complex with ACE2. At low ACE/Ang 2 levels, this complex is preserved, thereby providing normotension. In the case of hypertension, ACE increases and catalyzes the conversion of Ang 1 to Ang 2. The AT1R-ACE2 complex is impaired and ACE2 is released from AT1R and internalized into the cell; thus, vasoconstriction and fibrosis occur.

The RAAS system and its major enzymes ACE and ACE2 is also involved in the worsening of the clinical picture of COVID-19 infection [4]. The balance between ACE and ACE2 is disturbed in favor of ACE/Ang 2 in COVID-19 infection [5, 6]. ACE2 is also known to be a predominant receptor for SARS-CoV2 entry into the cell [2, 3]. The virus enters the cell by binding to ACE2, thereby letting AT1R alone and activating the ACE/Ang 2 pathway. Therefore, we hypothesized that ACE concentration may increase and, eventually leading to tissue damage in COVID-19 infection. Nevertheless, there are some conflicting data about ACE concentration/activity in COVID-19 disease [7–9].

Some risk factors determine the prognosis of COVID-19 infection, such as age, sex, concomitant diseases, levels of thyroid hormone, 25-OH-vitamin D and testosterone [10–13]. Insertion/deletion (I/D) polymorphism at intron 16 of the *ACE* gene was shown to be associated with ACE concentrations and hence the clinical severity of COVID-19 infection [14, 15]. ACE I/D polymorphism is correlated with the clinical severity of COVID-19 in different studies [14–18].

The coexistence of type 2 diabetes mellitus (T2DM) and hypertension (HT) also increased the mortality and severity of COVID-19 infection by altering the balance between the ACE1/Ang2 and ACE2 systems and the interaction of SARS-CoV-2 with the host [19–21].

Therefore, we aimed to investigate the association between ACE levels, *ACE* gene polymorphisms and the prognosis of COVID-19. We also aimed to evaluate the link between RAAS, T2DM and HT in COVID-19 patients.

## Materials and methods

### Selection of patients

This cross-sectional study was approved by the local Ethics Committee of Marmara University (11.05.2020, ethics no.: 536) and the Turkish Ministry of Health. Written informed consent was obtained from all participants. The study population consisted of individuals with reverse transcription polymerase chain reaction (RT‒PCR)-confirmed COVID-19 who were admitted to Marmara University Education & Research (E&R) Hospital between April and October 2020. We assessed these individuals for eligibility and included 266 individuals. The inclusion criteria were being aged >18 years and willing to participate in the study. Our exclusion criteria were not having a PCR, aged <18 years, ongoing renal or liver disease, cardiac or respiratory failure and malignancy, inability to access or missing electronic health records and refusal to participate in the study. The ACE gene polymorphism was analyzed in all individuals, and there were 208 individuals with ACE level results.

### Data collection

Demographic parameters (age and sex), symptoms at admission, clinical findings, vital signs, oxygen demand and thorax computed tomography (CT) findings were recorded. The duration of hospitalization, history of chronic illnesses and medications were also recorded.

The laboratory data (lymphocyte, neutrophil and thrombocyte counts, creatinine, alanine aminotransferase, high sensitive C‐reactive protein [hs-CRP], lactate dehydrogenase [LDH], ferritin, D‐dimer and procalcitonin values) were obtained on admission.

Blood samples obtained within 48 hours of admission to the hospital were centrifuged and serum aliquots were stored at −20°C for measurement of ACE levels. At the same time, 2 mL of EDTA anticoagulated blood was taken and stored for genomic DNA analysis. The neutrophil-lymphocyte ratio (NLR) was calculated by dividing the neutrophil count by the lymphocyte count. The findings observed on thorax computed tomography (CT) were also recorded.

### Evaluation of World Health Organization clinical severity scores

The patients were classified into 10 groups according to the COVID-19 severity index of the World Health Organization (WHO) working group. After the WHO classification, the patients were divided into four groups according to the WHO clinical progression scale: 1. mild ambulatory disease (1–3), 2. hospitalized with moderate disease (4–5), and 3. hospitalized with severe disease and dead (6–10). The need for noninvasive mechanical ventilation (NIMV), reservoir mask, admission to the ICU, mortality and WHO clinical progression scales were also reviewed [22].

### Biochemical measurements

Complete blood counts were measured with a Unicel DxH800 Coulter Cell Analyzer (Beckman Coulter) from K2EDTA samples. Serum LDH, creatinine and ALT were analyzed spectrophotometrically with AU 680 (Beckman Coulter). Ferritin levels were measured with a two‐site immunoenzymatic assay in Access Analyzer (Beckman Coulter). The D‐dimer parameter was quantitated with an immuno‐turbidimetric assay in 3.2% sodium citrated venous plasma (STA Compact, Diagnostica Stago). Hs-CRP levels were measured with a nephelometric method (BN Prospec, Dade Behring). The electrochemiluminescence immunoassay method was used in the measurement of procalcitonin (Cobas e‐411 Analyzer, Roche Diagnostics).

### ACE level measurement

Serum ACE levels were measured by ELISA (Elabscience, USA), which utilizes a sandwich ELISA principle. The samples were centrifuged for 15 min at 1000×g at 2–8°C and stored at -80°C until analysis. The detection range was between 0.78–50 ng/mL. As claimed by the manufacturer, the intra-assay precision ranges between 6.79–5.11% for a mean concentration between 2.65–21.79 ng/mL and the inter-assay precision range between 6.77–3.62% for a mean concentration between 2.51–22.37 ng/mL.

### I and D allele determination of the ACE 1 gene

D and I alleles in intron 16 of the *ACE* gene were determined on the basis of polymerase chain reaction (PCR) and the products were visualized by agarose gel electrophoresis. Genomic DNA was isolated from 5 mL of peripheral blood using the ZeeSan Lab-Aid® 824 sBlood DNA isolation kit. The quality and concentration of genomic DNA samples were measured by a NanoDrop (NanoDrop™ 2000/2000c spectrophotometers). For PCR, 100 ng genomic DNA was added to 10x PCR Buffer, 2 mM MgCl2, 0.5 mM dNTP, 0.1 pmol primers and 1 unit of *Thermus aquaticus* DNA polymerase with a total reaction volume of 25 μL. The amplicon sizes of the first primer pair for the detection of D and I alleles were 319 bp and 597 bp, respectively (hace3s, 5’, GCCCTGCAGGTGTCTGCAGCATGT3’; hace3as, 5’GGATGGCTCTCCCCGCCTTGTCTC3’). PCR (BIO-RAD T100) conditions included denaturation at 94°C for 30 seconds, annealing at 56°C for 45 seconds, and extension at 72°C for two minutes, repeated for 35 cycles, followed by a final extension at 72°C for seven minutes. PCR products were conducted at 98 V and 36 min. on 1.5% agarose gel electrophoresis. Amplicons were visualized by spectrophotometer (Gel Doc XR+ System, BIO-RAD). The second primer pair (hace5a, 5’TGGGACCACAGCGCCCGCCACTAC3’; hace5c, 5’TCGCCAGCCCTCCCATGCCCATAA3’) was used for the DD allele control due to the predominance of D allele amplifications in heterozygous samples. All samples that showed the DD allele pattern were amplified a second time with this primer pair. The PCR conditions were same as the previous conditions except for an annealing temperature of 67°C. PCR products that included the I allele had an amplicon size of 335-bp. The homozygous DD allele did not show any band under UV. We grouped the patients according to the determination of *ACE 1* alleles as DD, ID, or II.

### Categorical variables

The patients were grouped based on their medical history, including the presence of type 2 diabetes mellitus (T2DM), hypertension (HT), coronary artery disease (CAD), chronic obstructive pulmonary disease (COPD), asthma, or cerebrovascular accident (CVA). They were also grouped according to the use of dipeptidyl peptidase 4 enzyme inhibitor (DPP4i), ACE inhibitor (ACEi), or angiotensin receptor blocker (ARB).

### Statistical analysis

Data obtained in the study were analyzed statistically using SPSS 25.0 (IBM Corporation, Armonk, New York, United States). The conformity of the data to a normal distribution was evaluated using the Shapiro‒Wilk Francia test. Homogeneity of variance was evaluated with the Levene test. When comparing two independent groups of quantitative data, we used the independent-samples t test with bootstrap results or the Mann‒Whitney U test with the Monte Carlo simulation results. When comparing more than two groups of quantitative data according to each other, we used one-way ANOVA (robust test: Brown-Forsythe) and the Kruskal‒Wallis H test. Dunn’s and Tukey HSB tests were used for post hoc analyses. To analyze the correlations of the variables, Kendall’s tau-b test was used. When comparing categorical variables, the Pearson chi-square, Fisher exact and Fisher-Freeman-Halton tests with the Monte Carlo simulation technique were used. The comparison of column ratios was expressed by Benjamini-Hochberg corrected p values. To detect and estimate the most important variable, various machine learning methods incounseling were employed, including logistic regression, discriminant analysis, support vector machine, random forest, K-nearest neighbor algorithm, simple (Native) Bayes classification and neural network (multilayer perceptron radial basis). We utilized the results of neural network (multilayer perceptron) analysis, which provided the most successful model. We used gradient descent for the optimization algorithm, hyperbolic tangent and sigmoid for the hidden layer activation function and Softmax and Sigmoid for the output layer activation function. We used the mini-batch method to select the training data, adjusting it as 100% for the training set and 0% for the testing set. To detect the relationship between the real classification of the procedure’s success and the classification made by the cutoff values, the sensitivity and specificity ratios were expressed by ROC (receiver operating characteristic) curve analysis. Quantitative variables were reported as mean (standard deviation) and median (percentile 25/percentile 75) values, and categorical variables were stated as number (n) and percentage (%) in the tables. The variables were evaluated at a 95% confidence level and a value of p<0.05 was accepted as statistically significant.

## Results

Our study enrolled 266 patients in total. Their mean age was 57.6 (±14.2) years and 42.9% (n = 114) of them were female. Severe disease was found in 31.6% of the patients (n = 84). Of the patients, 33 (12.4%) were admitted to the ICU, and 11 (4.1%) died. Other clinical and laboratory findings of the patients are given in S1 Table.

Gene analysis detected DD polymorphisms in the ACE gene in 32.7% (n = 87) of the patients, ID in 51.5% (n = 137), and II in 15.8% (n = 42) (Table 1). Demographic, clinical and laboratory parameters were similar among different polymorphisms of the *ACE* gene (Table 1).

**Table 1:** Comparison of demographic, clinical and laboratory findings among different *ACE* gene polymorphisms.

	***ACE* gene**			**p**	**I allele**		**p**	**D allele**		**P**
	**DD (n = 87)**	**ID (n = 137)**	**II (n = 42)**		**Absent (n = 87)**	**Present (n = 179)**		**Absent (n = 42)**	**Present (n = 224)**	
	**Mean (SD.)**	**Mean (SD.)**	**Mean (SD.)**		**Mean (SD.)**	**Mean (SD.)**		**Mean (SD.)**	**Mean (SD.)**	
**Age (year)**	56.6 (12.8	58.1 (15.6)	57.7 (12.3)	0.714 ^a^	56.6 (12.8)	582 (14.9)	0.429 ^t^	57.7 (12.3)	57.5 (14.6)	0.923 ^t^
	**n(%)**	**n(%)**	**n(%)**		**n(%)**	**n(%)**		**n(%)**	**n(%)**	
**Sex (Female)**	36 (41.4)	58 (42.3)	20 (47.6)	0.787 ^c^	36 (41.4)	78 (43.6)	0.792 ^c^	20 (47.6)	94 (42)	0.304 ^c^
**Exitus**	2 (2.3)	8 (5.8)	1 (2.4)	0.483 ^ff^	2 (2.3)	9 (5)	0.512 ^f^	1 (2.4)	10 (4.5)	0.458 ^f^
**T2DM**	29 (33.3)	38 (27.7)	8 (19)	0.236 ^c^	29 (33.3)	46 (25.7)	0.245 ^c^	8 (19)	67 (29.9)	0.103 ^c^
**HT**	39 (44.8)	57 (41.6)	20 (47.6)	0.776 ^c^	39 (44.8)	77 (43)	0.793 ^c^	20 (47.6)	96 (42.9)	0.343 ^c^
**CAD**	15 (17.2)	18 (13.1)	5 (11.9)	0.626 ^c^	15 (17.2)	23 (12.8)	0.354 ^c^	5 (11.9)	33 (14.7)	0.420 ^c^
**COPD/Asthma**	6 (6.9)	18 (13.1)	4 (9.5)	0.351 ^ff^	6 (6.9)	22 (12.3)	0.207 ^c^	4 (9.5)	24 (10.7)	0.537 ^f^
**CVA**	3 (3.4)	2 (1.5)	2 (4.8)	0.277 ^ff^	3 (3.4)	4 (2.2)	0.686 ^f^	2 (4.8)	5 (2.2)	0.305 ^f^
**ACEi/ARB**	27 (31)	35 (25.5)	12 (28.6)	0.671 ^c^	27 (31)	47 (26.3)	0.466 ^c^	12 (28.6)	62 (27.7)	0.520 ^c^
**DPP4i**	9 (10.3)	7 (5.1)	0 (0)	0.058 ^ff^	9 (10.3)	7 (3.9)	0.053 ^c^	0 (0)	16 (7.1)	0.059 ^f^
**CT findings**	81 (93.1)	119 (86.9)	36 (85.7)	0.269 ^ff^	81 (93.1)	155 (86.6)	0.148 ^c^	36 (85.7)	200 (89.3)	0.328 ^f^
**Oxygen demand**	58 (66.7)	90 (65.7)	25 (59.5)	0.702 ^c^	58 (66.7)	115 (64.2)	0.784 ^c^	25 (59.5)	148 (66.1)	0.259 ^c^
**ICU admission**	11 (12.6)	16 (11.7)	6 (14.3)	0.909 ^c^	11 (12.6)	22 (12.3)	0.999 ^c^	6 (14.3)	27 (12.1)	0.424 ^c^
**Disease severity**				0.442 ^c^			0.143 ^c^			0.852 ^c^
Mild	8 (9.2)	19 (13.9)	6 (14.3)		8 (9.2)	25 (14)		6 (14.3)	27 (12.1)	
Moderate	56 (64.4)	71 (51.8)	22 (52.4)		56 (64.4)	93 (52)		22 (52.4)	127 (56.7)	
Severe	23 (26.4)	47 (34.3)	14 (33.3)		23 (26.4)	61 (34.1)		14 (33.3)	70 (31.3)	
	**Median (Q1/Q3)**	**Median (Q1/Q3)**	**Median (Q1/Q3)**		**Median (Q1/Q3)**	**Median (Q1/Q3)**		**Median (Q1/Q3)**	**Median (Q1/Q3)**	
**Duration of hospitalization(day)**	10 (7/16)	10 (7/16)	12 (6/17)	0.974 ^k^	10 (7/16)	11 (7/16)	0.852 ^u^	12 (6/17)	10 (7/16)	0.948 ^u^
**Respiratory rate(/min)**	24 (20/30)	22 (20/28)	22 (20/28)	0.630 ^k^	24 (20/30)	22 (20/28)	0.575 ^u^	22 (20/28)	23.5 (20/28)	0.367 ^u^
**Lymphocyte(/mm** ^**3**^ **)**	1000 (700/1400)	1000 (800/1400)	1000 (700/1500)	0.945 ^k^	1000 (700/1400)	1000 (800/1400)	0.824 ^u^	1000 (700/1500)	1000 (750/1400)	0.760 ^u^
**Ferritin (mcg/L)**	184 (67.7/435)	172 (75.6/410.7)	198.3 (88.4/381.6)	0.983 ^k^	184 (67.7/435)	186.5 (86.5/401)	0.868 ^u^	198.3 (88.4/381.6)	181 (72.5/427)	0.918 ^u^
**LDH (U/L)**	267 (213/372)	271 (217/342)	270 (202/433)	0.883 ^k^	267 (213/372)	271 (211/352)	0.621 ^u^	270 (202/433)	269.5 (216/350.5)	0.809 ^u^
**Hs-CRP (mg/dL)**	31.6 (11.2/102.8)	38.8 (11.1/78)	24.7 (11.4/93.7)	0.815 ^k^	31.6 (11.2/102.8)	36.1 (11.1/81)	0.667 ^u^	24.7 (11.4/93.7)	37.8 (11.2/96.7)	0.560 ^u^
**D-dimer (mcg/mL)**	0.6 (0.4/0.9)	0.7 (0.4/1.2)	0.6 (0.4/1)	0.196 ^k^	0.6 (0.4/0.9)	0.7 (0.4/1.2)	0.166 ^u^	0.6 (0.4/1)	0.6 (0.4/1.1)	0.513 ^u^
**Procalcitonin (ng/mL)**	0.1 (0.1/0.2)	0.1 (0.1/0.1)	0.1 (0.1/0.2)	0.769 ^k^	0.1 (0.1/0.2)	0.1 (0.1/0.1)	0.627 ^u^	0.1 (0.1/0.2)	0.1 (0.1/0.1)	0.738 ^u^
**ACE level (ng/mL)**	19.2 (13.0 / 26.5)	20.0 (12.2 / 27.2)	17.8 (12.4 / 26.5)	0.725 ^k^	19.2 (13.0 / 26.5)	19.0 (12.3 / 27.2)	0.967 ^u^	17.8 (12.4 / 26.5)	19.5 (12.5 / 26.8)	0.453 ^u^

T2DM, type 2 diabetes mellitus; HT, hypertension; CAD, coronary artery disease; COPD, chronic obstructive pulmonary disease; CVA, cerebrovascular accident; ACEi, angiotensin converting enzyme inhibitor; ARB, angiotensin II receptor blocker; DPP4i, dipeptidyl peptidase-4 inhibitor; CT, computed tomography; ICU, intensive care unit; LDH, lactate dehydrogenase; hs-CRP, high sensitive C‐reactive protein; ACE, angiotensin converting enzyme.

^k^ Kruskal Wallis Test (Monte Carlo)

^t^ Independent t Test (Bootstrap)

^u^ Mann-Whitney U Test (Monte Carlo)

^ff^ Fisher Freeman Halton (Monte Carlo)

^f^ Fisher Exact Test (Monte Carlo)

^c^ Pearson Chi Square Test (Monte Carlo); SD., Standard deviation; Q1, 1st Quartile; Q3, 3th Quartile.

The mean serum ACE level was 21.3 (±11.2) ng/dL. ACE levels were higher in patients who died during COVID-19 (29.6 ng/mL vs 17.4 ng/mL, p = 0.023) and were admitted to the ICU (37.2 ng/mL vs 16.7 ng/mL, p<0.001). ACE levels were higher in severe disease (25.7 ng/mL) than in mild (17.1 ng/mL, p = 0.023) and moderate (16.3 ng/mL, p<0.001) cases, but the difference was not significant between mild and moderate cases. ACE levels were positively correlated with age (p<0.001), duration of hospitalization (p = 0.012), serum ferritin (p<0.001), hs-CRP (p = 0.001), procalcitonin (p = 0.002), D dimer (p<0.001) levels and LDH activity (p<0.001) (Table 2).

**Table 2:** Comparison of ACE level of the patients with different demographic and clinical findings, and correlation of ACE levels with other parameters*.

**(n = 159)**	**ACE level Median (Q1/Q3)**				**p**
**Sex (Female—Male)**	17.7 (11.9/26.2)		20 (13.7/27.5)		0.125 ^u^
	**Absent**		**Present**		
**Mortality**	17.4 (11.5 / 26.3)		29.6 (24.8 / 41.7)		**0.023** ^u^
**T2DM**	16.7 (11.4 / 26.5)		19.0 (15.5 / 26.3)		0.143 ^u^
**HT**	17.3 (11.5 / 25.9)		19.1 (13.8 / 33.4)		0.131 ^u^
**CAD**	18.2 (11.9 / 26.7)		14.7 (10.6 / 20.3)		0.144 ^u^
**COPD/Asthma**	17.4 (11.8 / 26.1)		19.0 (10.8 / 27.2)		0.832 ^u^
**CVA**	18.1 (11.8 / 26.5)		11.8 (9.6 / 13.8)		0.111 ^u^
**DPP4i**	17.4 (11.5 / 26.3)		22.5 (18.6 / 37.3)		0.112 ^u^
**CT findings**	16.1 (8.6 / 19.0)		18.3 (11.8 / 26.7)		0.089 ^u^
**Oxygen Demand**	15.7 (10.8 / 22.0)		18.3 (12.5 / 27.3)		0.062 ^u^
**ICU admission**	16.7 (11.5 / 24.2)		37.2 (18.9 / 43.9)		**<0.001** ^u^
	**Mild**	**Moderate**		**Severe**	
**Disease severity**	17.1 (11.5 / 21.7)	16.3 (10.8 / 22.0)		25.7 (15.7 / 37.1) **^A^^B^**	**0.001 ^j^**
**CORRELATIONS**					
		**r**			**P**
**Age**		0,195			**<0.001 ^b^**
**Duration of hospitalization(day)**		0,137			**0.012 ^b^**
**Respiration rate (/min)**		0,061			0.276 **^b^**
**Lymphocyte (/mm** ^**3**^ **)**		-0,077			0.159 **^b^**
**Lymphocyte (%)**		-0,062			0.248 **^b^**
**Neutrophil (/mm** ^**3**^ **)**		0,037			0.493 **^b^**
**Neutrophil (%)**		0,037			0.492 **^b^**
**NLR**		0,083			0.123 **^b^**
**Platelet (/mm** ^**3**^ **)**		0,037			0.485 **^b^**
**Ferritin (mcg/L)**		0,241			**<0.001 ^b^**
**LDH (U/L)**		0,190			**<0.001 ^b^**
**Hs-CRP (mg/dL)**		0,177			**0.001 ^b^**
**D-dimer (mcg/mL)**		0,193			**<0.001 ^b^**
**Procalcitonin (ng/mL)**		0,167			**0.002 ^b^**

ACE, angiotensin converting enzyme; T2DM, type 2 diabetes mellitus; HT, hypertension; CAD, coronary artery disease; COPD, chronic obstructive pulmonary disease; CVA, cerebrovascular accident; ACEi, angiotensin converting enzyme inhibitor; ARB, angiotensin II receptor blocker; DPP4i, dipeptidyl peptidase-4 inhibitor; CT, computed tomography; ICU, intensive care unit; NLR, neutrophil/lymphocyte ratio; LDH, lactate dehydrogenase; hs-CRP, high sensitive C‐reactive protein.

^u^ Mann-Whitney U Test (Monte Carlo)

^b^ Kendall’s tau b Test, SD., Standard deviation

^j^ Jonckheere-Terpstra Test (Monte Carlo); Q1, 1st Quartile; Q3, 3th Quartile

^A^ Significance comparing mild

^B^ Significance comparing moderate group.

* Patients using ACEi/ARB were excluded from the analysis.

The mean age (46.3 years) was the lowest in the mild disease group (p<0.001). Death was detected only in the severe disease group. One patient with moderate disease and 32 patients with severe disease were admitted to the ICU (p<0.001). The presence of T2DM (p = 0.001), HT (p<0.001), and CAD (p = 0.016) and the use of ACEi/ARB (p = 0.003) were less frequent in the mild disease group than in either moderate or severe cases. DPP4i use was similar among different groups of disease severity. The duration of hospitalization (6 vs 18 days, p<0.001) and the respiratory rate (20 vs 28/min; p<0.001) were the highest in the severe disease group compared to mild cases. A comparison of other findings according to disease severity is given in Table 3.

**Table 3:** Comparison of demographic, clinical and laboratory findings according to disease severity.

	Disease severity			P	Pairwise comparison		
	Mild (n = 33)	Moderate (n = 149)	Severe (n = 84)		Mild vs Moderate	Mild vs Severe	Moderate vs Severe
	**Mean (SD.)**	**Mean (SD.)**	**Mean (SD.)**				
**Age**	46.3 (13.3)	59.3 (13.5)	59.0 (13.9)	**<0.001 ^a^**	**<0.001**	**<0.001**	**0.987**
	**n(%)**	**n(%)**	**n(%)**				
**Sex (Female)**	17 (51.5)	69 (46.3)	28 (33.3)	0.093 **^c^**	ns.	ns.	ns.
**Exitus**	0 (0)	0 (0)	11 (13.1)	**<0.001 ^ff^**	ns.	**<0.001**	**<0.001**
**T2DM**	2 (6.1)	48 (32.2)	25 (29.8)	**0.010 ^c^**	**0.007**	**0.009**	ns.
**HT**	3 (9.1)	73 (49)	40 (47.6)	**<0.001 ^c^**	**<0.001**	**<0.001**	ns.
**CAD**	0 (0)	26 (17.4)	12 (14.3)	**0.016 ^ff^**	**0.001**	**0,001**	ns.
**COPD/Asthma**	3 (9.1)	15 (10.1)	10 (11.9)	0.919 **^ff^**	ns.	ns.	ns.
**CVA**	0 (0)	4 (2.7)	3 (3.6)	0.673 **^c^**	ns.	ns.	ns.
**ACEi/ARB**	1 (3)	49 (32.9)	24 (28.6)	**0.003 ^c^**	**0.002**	**0.004**	ns.
**DPP4i**	0 (0)	10 (6.7)	6 (7.1)	0.371 **^ff^**	ns.	ns.	ns.
**CT findings**	20 (60.6)	133 (89.3)	83 (98.8)	**<0.001 ^ff^**	**<0.001**	**<0.001**	0.007
**ICU admission**	0 (0)	1 (0.7)	32 (38.1)	**<0.001 ^ff^**	ns.	ns.	**<0.001**
**Oxygen demand**	1 (3)	88 (59.1)	84 (100)	**<0.001 ^c^**	**<0.001**	**<0.001**	ns.
	**Median (Q1/Q3)**	**Median (Q1/Q3)**	**Median (Q1/Q3)**				
**Duration of hospitalization (day)**	6 (5/7)	9 (7/12)	18 (13/27.5)	**<0.001 ^k^**	**<0.001**	**<0.001**	**<0.001**
**Respiratory rate (/min)**	20 (20/24)	22 (20/26)	28 (22/32)	**<0.001 ^k^**	**0.017**	**<0.001**	**<0.001**
**Lymphocyte (/mm** ^**3**^ **)**	1200 (900/1500)	1100 (800/1400)	800 (600/1300)	**0.003 ^k^**	**0.999**	**0.015**	**0.009**
**Lymphocyte (%)**	23.4 (17.9/30)	20.3 (14.8/29)	12.9 (9.2/20.4)	**<0.001 ^k^**	**0.798**	**<0.001**	**<0.001**
**Neutrophil (/mm** ^**3**^ **)**	3000 (2500/4300)	3600 (2700/5100)	4500 (3400/6850)	**<0.001 ^k^**	**0.843**	**0.001**	**<0.001**
**Neutrophil (%)**	65.9 (59.1/70.6)	69.2 (60.2/75.2)	77.4 (69.3/83.2)	**<0.001 ^k^**	**0.284**	**<0.001**	**<0.001**
**NLR**	2.8 (1.9/3.9)	3.5 (2.1/5.2)	5.9 (3.3/8.9)	**<0.001 ^k^**	**0.614**	**<0.001**	**<0.001**
**Platelet (/mm** ^**3**^ **)**	207 (165/250)	180 (146/226)	187 (143.5/271.5)	0.265 **^k^**	ns.	ns.	ns.
**Ferritin (mcg/L)**	128.9 (75.2/244)	161.3 (60.6/357)	283.8 (128/728.5)	**<0.001 ^k^**	**0.999**	**0.006**	**<0.001**
**LDH (U/L)**	267 (202/334)	248 (204/309)	321 (233/484)	**<0.001 ^k^**	0.999	**0.028**	**<0.001**
**Hs-CRP (mg/dL)**	12 (6.6/39.9)	25 (9.4/71)	80.2 (33.8/156)	**<0.001 ^k^**	**0.421**	**<0.001**	**<0.001**
**D-dimer (mcg/mL)**	0.5 (0.3/0.6)	0.6 (0.4/0.9)	0.9 (0.6/1.6)	**<0.001 ^k^**	**0.356**	**<0.001**	**<0.001**
**Procalcitonin (ng/mL)**	0.1 (0.1/0.1)	0.1 (0.1/0.1)	0.1 (0.1/0.2)	**<0.001 ^k^**	**0,999**	**<0.001**	**<0.001**

T2DM, type 2 diabetes mellitus; HT, hypertension; CAD, coronary artery disease; COPD, chronic obstructive pulmonary disease; CVA, cerebrovascular accident; ACEi, angiotensin converting enzyme inhibitör; ARB, angiotensin II receptor blocker; DPP4i, dipeptidyl peptidase-4 inhibitor; CT, computed tomography; ICU, intensive care unit; NLR, neutrophil/lymphocyte ratio; LDH, lactate dehydrogenase; hs-CRP, high sensitive C‐reactive protein.

^k^ Kruskal Wallis Test (Monte Carlo), Post Hoc Test, Dunn’s Test

^a^ OneWay ANOVA (Robuts Statistic, Brown-Forsythe), Post Hoc Test, Tukey HSD

^ff^ Fisher Freeman Halton (Monte Carlo), Post Hoc Test, Benjamini-Hochberg correction

^c^ Pearson Chi Square Test (Monte Carlo), Post Hoc Test, Benjamini-Hochberg correction; SD., Standard deviation; Q1, 1st Quartile; Q3, 3th Quartile; ns, not significant.

Disease severity (p<0.001) and oxygen demand (p = 0.009) were associated with mortality. Additionally, disease severity (p<0.001), oxygen demand (p<0.001), CT findings (p = 0.034) and respiratory rate (p<0.001) were associated with ICU admission. ACEi/ARB or DPP4i use was not associated with mortality or ICU admission. ACE levels were higher in the patients who died (18.6 vs 29.8; p = 0.004). ACE levels were also higher in the patients who were admitted to the ICU (18.2 vs 37.2 ng/mL; p<0.001) (Table 4).

**Table 4:** Comparison of demographic, clinical and laboratory parameters according to mortality or ICU admission.

		Mortality		p	Intensive Care Unit		p
		Absent (n = 255)	Present (n = 11)		Absent (n = 233)	Present (n = 33)	
		**Mean (SD.)**	**Mean (SD.)**		**Mean (SD.)**	**Mean (SD.)**	
**Age**		57.4 (14.1)	60.3 (16.6)	0.550 ^t^	57.5 (14.7)	58.2 (10.2)	0.726 ^t^
		**n (%)**	**n (%)**		**n (%)**	**n (%)**	
**Sex (Female)**		109 (42.7)	5 (45.5)	0.999 **^f^**	106 (45.5)	8 (24.2)	**0.024 ^c^**
**Disease severity**				**<0.001 ^ff^**			**<0.001 ^c^**
Mild		33 (12.9)	0 (0)		33 (14.2)	0 (0)	
Moderate		149 (58.4)	0 (0)		148 (63.5)	1 (3)	
Severe		73 (28.6)	11 (100)		52 (22.3)	32 (97)	
**T2DM**		70 (27.5)	5 (45.5)	0.302 **^f^**	62 (26.6)	13 (39.4)	0.148 **^c^**
**HT**		110 (43.1)	6 (54.5)	0.541 **^f^**	101 (43.3)	15 (45.5)	0.853 **^c^**
**CAD**		37 (14.5)	1 (9.1)	0.999 **^f^**	34 (14.6)	4 (12.1)	0.999 **^f^**
**COPD/Asthma**		26 (10.2)	2 (18.2)	0.325 **^f^**	24 (10.3)	4 (12.1)	0.762 **^f^**
**CVA**		6 (2.4)	1 (9.1)	0.259 **^f^**	4 (1.7)	3 (9.1)	**0.043 ^f^**
**ACEi/ARB**		70 (27.5)	4 (36.4)	0.505 **^f^**	66 (28.3)	8 (24.2)	0.684 **^c^**
**DPP4i**		15 (5.9)	1 (9.1)	0.501 **^f^**	12 (5.2)	4 (12.1)	0.121 **^f^**
**CT findings**		225 (88.2)	11 (100)	0.619 **^f^**	203 (87.1)	33 (100)	**0.034 ^f^**
**Oxygen demand**		162 (63.5)	11 (100)	**0.009 ^f^**	140 (60.1)	33 (100)	**<0.001 ^c^**
		**Median (Q1/Q3)**	**Median (Q1/Q3)**		**Median (Q1/Q3)**	**Median (Q1/Q3)**	
**Respiratory rate (/min)**		22 (20/28)	30 (24/32)	**0.003 ^u^**	22 (20/27)	30 (24/35)	**<0.001 ^u^**
**Lymphocyte (/mm** ^**3**^ **)**		1000 (800/1400)	800 (700/900)	0.101 **^u^**	1000 (800/1400)	700 (600/1200)	**0.001 ^u^**
**Lymphocyte (%)**		19.1 (12.9/27.8)	11.5 (9.1/13.8)	**<0.001 ^u^**	20.1 (13.8/28.2)	10.3 (8.5/16.5)	**<0.001 ^u^**
**Neutrophil (/mm** ^**3**^ **)**		3700 (2700/5200)	6700 (4700/9100)	**0.002 ^u^**	3600 (2700/4900)	6200 (4200/8800)	**<0.001 ^u^**
**Neutrophil (%)**		70.8 (61.4/78)	80.2 (75.6/83.2)	**0.002 ^u^**	69.4 (60.6/76.3)	80.3 (76.6/85.1)	**<0.001 ^u^**
**NLR**		3.7 (2.2/6.1)	6.7 (4.8/10)	**0.004 ^u^**	3.5 (2.2/5.6)	7.6 (4.6/11)	**<0.001 ^u^**
**Platelet (/mm** ^**3**^ **)**		182 (145/236)	196 (144/323)	0.288 **^u^**	182 (144/235)	192 (150/271)	0.205 **^u^**
**Ferritin (mcg/L)**		181.9 (75.4/410.7)	308.4 (147/968)	0.092 **^u^**	164.6 (70/372)	497 (205/985.2)	**<0.001 ^u^**
**LDH (U/L)**		267 (209/351)	320 (284/589)	**0.014 ^u^**	257 (205/327)	377 (300/538)	**<0.001 ^u^**
**Hs-CRP (mg/dL)**		32.4 (10.9/87.7)	183 (61.1/225.1)	**<0.001 ^u^**	27.4 (10.4/76.3)	116.7 (70.3/183)	**<0.001 ^u^**
**D-dimer (mcg/mL)**		0.6 (0.4/1)	1.7 (0.9/3.9)	**<0.001 ^u^**	0.6 (0.4/0.9)	1.3 (0.8/1.7)	**<0.001 ^u^**
**Procalcitonin (ng/mL)**		0.1 (0.1/0.1)	0.2 (0.1/0.6)	**0.001 ^u^**	0.1 (0.1/0.1)	0.1 (0.1/0.3)	**<0.001 ^u^**
**ACE level(ng/mL)**		18.6 (12.4 / 26.6)	29.8 (24.8 / 36.6)	**0.004 ^u^**	18.2 (11.9 / 25.9)	37.2 (19.4 / 43.9)	**<0.001 ^u^**

T2DM, type 2 diabetes mellitus; HT, hypertension; CAD, coronary artery disease; COPD, chronic obstructive pulmonary disease; CVA, cerebrovascular accident; ACEi, angiotensin converting enzyme inhibitor; ARB, angiotensin II receptor blocker; DPP4i, dipeptidyl peptidase-4 inhibitor; CT, computed tomography; NLR, neutrophil/lymphocyte ratio; LDH, lactate dehydrogenase; hs-CRP, high sensitive C‐reactive protein.

^t^ Independent t Test (Bootstrap)

^u^ Mann-Whitney U Test (Monte Carlo)

^ff^ Fisher Freeman Halton (Monte Carlo)

^f^ Fisher Exact Test (Monte Carlo)

^c^ Pearson Chi Square Test (Monte Carlo); SD., Standard deviation; Q1, 1st Quartile; Q3, 3th Quartile.

**Fig 1:**
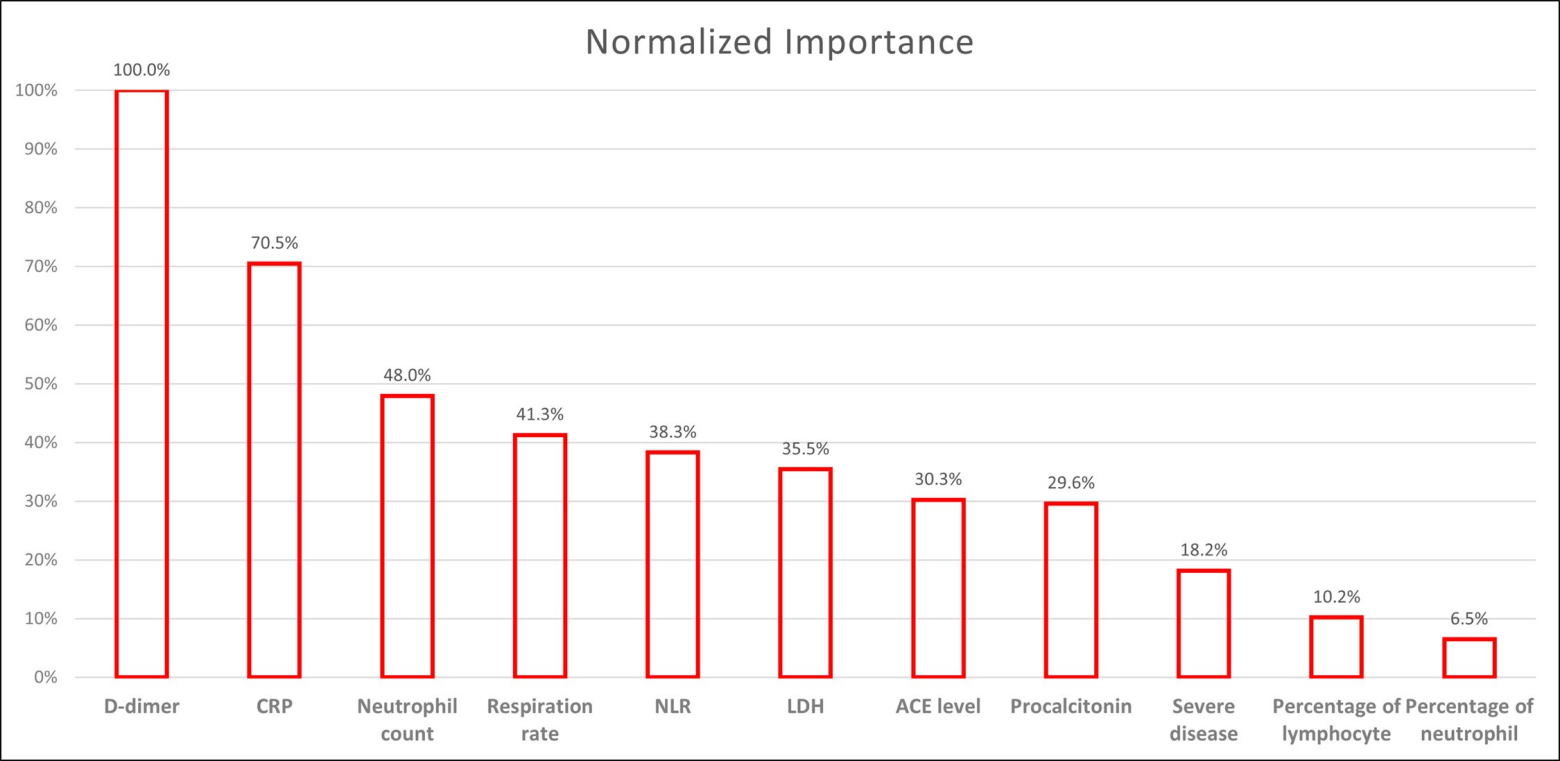
Prediction of Mortality. CRP, C-reactive protein; NLR, neutrophil/lymphocyte ratio; LDH, lactate dehydrogenase; ACE, angiotensin converting enzyme.

D-dimer and hs-CRP were the most important factors predicting mortality, with a normalized importance ratios of 100% and 70.5%, respectively. ACE level was a weak predictor of mortality with a normalized importance ratio of 30.3% (Fig 1).

**Fig 2:**
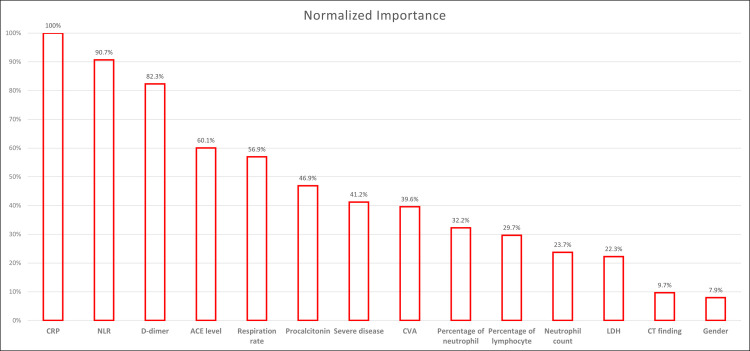
Prediction of Intensive Care Unit Admision. CRP, C-reactive protein; NLR, neutrophil/lymphocyte ratio; ACE, angiotensin converting enzyme; CVA, cerebrovascular accident; LDH, lactate dehydrogenase.

Hs-CRP, neutrophil to lymphocyte ratio (NLR), D-dimer, ACE level, and respiratory rate were important factors predicting ICU admission, with normalized importance ratios of 100, 90.7, 82.3, 60.1, and 56.9%, respectively (Fig 2).

Mortality or ICU admission were not associated with the presence of HT or ACEi/ARB use. Mild disease was more frequent in patients without HT (p<0.001). The ACE level of hypertensive patients treated with ACEi/ARB was higher (22.4 ng/mL) than in those without HT (17.3 ng/mL, p = 0.008) (Table 5). Mortality or ICU admission were not associated with the presence of T2DM or DPP-4i use. ACE levels were higher (21.8 ng/mL) in patients with T2DM treated with DPP4i than in those without T2DM (18 ng/mL, p = 0.049) (Table 6).

**Table 5:** Comparison of demographic, clinical and laboratory findings according to presence of HT and/or ACEi/ARB use.

	HT (-) (I)	HT (+) ACEi/ARB (+) (II)	HT (+) ACEi/ARB (-) (III)	p	Pairwise comparison		
	(n = 148)	(n = 74)	(n = 44)		I vs II	I vs III	II vs III
	**Mean (SD.)**	**Mean (SD.)**	**Mean (SD.)**				
**Age**	51.2 (13)	66.4 (11.7)	64 (10.8)	**<0.001 ^a^**	**<0.001**	**<0.001**	**0.557**
	**n(%)**	**n(%)**	**n(%)**				
**Sex (Female)**	54 (36.5)	42 (56.8)	18 (40.9)	**0.016** ^c^	**0.012**	ns.	ns.
**Disease severity**				**<0.001** ^c^			
Mild	30 (20.3)	1 (1.4)	2 (4.5)		**<0.001**	ns.	**0.021**
Moderate	75 (50.7)	49 (66.2)	25 (56.8)		ns.	ns.	ns.
Severe	43 (29.1)	24 (32.4)	17 (38.6)		ns.	ns.	ns.
**Mortality**	4 (2.7)	4 (5.4)	3 (6.8)	0.338 ^ff^	ns.	ns.	ns.
**ICU admission**	17 (11.5)	8 (10.8)	8 (18.2)	0.486 ^c^	ns.	ns.	ns.
**CT findings**	130 (87.8)	68 (91.9)	38 (86.4)	0.605 ^ff^	ns.	ns.	ns.
**Oxygen demand**	95 (64.2)	45 (60.8)	33 (75)	0.296 ^c^	ns.	ns.	ns.
	**Median (Q1/Q3)**	**Median (Q1/Q3)**	**Median (Q1/Q3)**				
**ACE (ng/mL)**	17.3 (11.5/25.9)	22.4 (18.5/29.1)	19.1 (13.8/33.4)	**0.005** ^k^	**0,008**	0,374	0,999
**Duration of hospitalization (day)**	10 (6.5/16)	9.5 (7/15)	12 (8.5/20)	0.090 ^k^	ns.	ns.	ns.
**Respiratory rate(/min)**	22 (20/29)	22 (20/28)	24 (22/30)	0.128 ^k^	ns.	ns.	ns.

HT, hypertension; ACEi, angiotensin converting enzyme inhibitor; ARB, angiotensin II receptor blocker; ICU, intensive care unit; CT, computed tomography; ACE, angiotensin converting enzyme.

^k^ Kruskal Wallis Test (Monte Carlo), Post Hoc Test, Dunn’s Test

^a^ OneWay ANOVA (Robuts Statistic, Brown-Forsythe), Post Hoc Test, Tukey HSD

^ff^ Fisher Freeman Halton (Monte Carlo)

^c^ Pearson Chi Square Test (Monte Carlo), Post Hoc Test, Benjamini-Hochberg correction; SD., Standard deviation; Q1, 1st Quartile; Q3, 3th Quartile; ns., not significant.

**Table 6:** Comparison of demographic, clinical and laboratory findings according to presence of T2DM and/or DPP4i use.

	T2DM (-) (I)	T2DM (+) DPP4i (+) (II)	T2DM (+) DPP4i (-) (III)	p	Pairwise comparison		
	(n = 191)	(n = 16)	(n = 59)		I vs II	I vs III	II vs III
	**Mean (SD.)**	**Mean (SD.)**	**Mean (SD.)**				
**Age**	56 (14.7)	58.4 (10.5)	62.3 (12.5)	**0.011 ^a^**	0.798	**0.009**	0.588
	**n(%)**	**n(%)**	**n(%)**				
**Sex (Female)**	75 (39.3)	5 (31.3)	34 (57.6)	**0.029 ^c^**	ns.	**0.039**	ns.
**Disease severity**				**0.038** ^ff^			
Mild	31 (16.2)	0 (0)	2 (3.4)		ns.	**0.011**	ns.
Moderate	101 (52.9)	10 (62.5)	38 (64.4)		ns.	ns.	ns.
Severe	59 (30.9)	6 (37.5)	19 (32.2)		ns.	ns.	ns.
**Mortality**	6 (3.1)	1 (6.3)	4 (6.8)	0.303 ^ff^	ns.	ns.	ns.
**ICU admission**	20 (10.5)	4 (25)	9 (15.3)	0.151 ^ff^	ns.	ns.	ns.
**CT findings**	166 (86.9)	16 (100)	54 (91.5)	0.282 ^ff^	ns.	ns.	ns.
**Oxygen demand**	120 (62.8)	13 (81.3)	40 (67.8)	0.292 ^c^	ns.	ns.	ns.
	**Median (Q1/Q3)**	**Median (Q1/Q3)**	**Median (Q1/Q3)**				
**ACE (ng/mL)**	18 (11.8/26.3)	21.8 (19.6/37.3)	21 (15.7/29.4)	**0.040** ^k^	**0.049**	0.062	0.427
**Duration of hospitalization (days)**	10 (7/16)	10.5 (6.5/17.5)	11 (7/15)	0.994 ^k^	ns.	ns.	ns.
**Respiratory rate (/min)**	22 (20/28)	25 (21.5/30)	24 (21/28)	0.327 ^k^	ns.	ns.	ns.

T2DM, type 2 diabetes mellitus; DPP4i, dipeptidyl peptidase-4 inhibitor; ICU, intensive care unit; CT, computed tomography; ACE, angiotensin converting enzyme.

^k^ Kruskal Wallis Test (Monte Carlo), Post Hoc Test, Dunn’s Test

^a^ OneWay ANOVA (Robuts Statistic, Brown-Forsythe), Post Hoc Test, Tukey HSD

^ff^ Fisher Freeman Halton (Monte Carlo), Post Hoc Test, Benjamini-Hochberg correction

^c^ Pearson Chi Square Test (Monte Carlo), Post Hoc Test, Benjamini-Hochberg correction; SD., Standard deviation; Q1, 1^st^ Quartile; Q3, 3th Quartile; ns., not significant.

In total, an ACE level of >37.092 ng/mL predicted ICU admission with a sensitivity of 92.5% (AUC = 0.775, p<0.001). ACE levels of >26.112 ng/mL and >26.098 ng/mL predicted ICU admission with a sensitivity of 70% in patients without HT and in those without T2DM (AUC = 0.777, p = 0.004; and AUC = 0.768, p = 0.005), respectively. In patients with HT not treated with ACEi/ARB, ACE level of >18.553 ng/mL predicted ICU admission with a sensitivity of 100% (AUC = 0.779, p = 0.049) (Table 7).

**Table 7:** ROC analysis showing cutoff values for ACE levels predicting ICU admission in different patient groups.

	ICU admission		ACE level cutoff	Sensitivity	Specificity	AUC (SE.)	p
	Absent	Present					
**HT (-)**	131	17	26.1125	70.0%	80.0%	0.777 (0.084)	**0.004**
**HT (+) ACEi/ARB (+)**	66	8	38.7225	50.0%	93.0%	0.705 (0.124)	0.106
**HT (+) ACEi/ARB (-)**	36	8	18.553	100.0%	51.7%	0.779 (0.092)	**0.049**
**T2DM (-)**	171	20	26.098	70.0%	77.7%	0.768 (0.086)	**0.005**
**T2DM (+) DPP4i (-)**	50	9	17.9445	100.0%	41.0%	0.634 (0.096)	0.264
**Total**	233	33	37.092	92.5%	52.4%	0.775 (0.053)	**<0.001**

ICU, intensive care unit; HT, hypertension; ACEi, angiotensin converting enzyme inhibitor; ARB, angiotensin II receptor blocker; T2DM, type 2 diabetes mellitus; DPP4i, dipeptidyl peptidase-4 inhibitor.

Roc, (Receiver Operating Curve) Analysis (Honley&Mc Nell–Youden index J); AUC, Area under the ROC curve; SE.,standard error.

## Discussion

Based on the results, our study suggested that *ACE* gene polymorphism was not associated with ACE level and disease severity, ICU admission, or mortality in COVID-19 disease. Remarkably, ACE levels were higher in patients with severe disease. Moreover, ACE levels were higher in patients who were admitted to the ICU and died due to COVID-19. Considering COVID-19 together with chronic diseases and their treatments, ACE levels were similar between the patients with T2DM treated with or without DPP-4i. It was also similar between the patients with HT treated with or without ACEi/ARB.

In our study, the DD polymorphism was present in approximately one-third of the patients, whereas more than half of the patients exhibited the ID genotype for the *ACE* gene. Ethnic differences were found to be a significant factor contributing to *ACE* gene polymorphism [14, 17]. The frequency of insertion and deletion was to be 41.2% and 58.8% in Europe, and 36% and 64% in the Middle East, respectively [14]. The different clinical courses of COVID-19 among various populations may be associated, at least in part, with *ACE* gene polymorphisms. In one study from Turkey, including 90 patients with COVID-19, the DD genotype was present in half of the patients, the II genotype in one-third, and the I/D genotype in the others. In this study, ACE II was the predominant genotype in asymptomatic patients, while ACE DD was the predominant genotype in the severe disease group. This study also reported an association between *ACE* gene polymorphism and disease severity, where the ACE II genotype was found to be protective against severe COVID-19. Another study showed that the *ACE* gene D allele was associated with more severe SARS-CoV-2 infection [23]. The ACE gene D allele is also involved in RAAS-associated pathological process [24]. Although many studies have shown an association of the ACE DD genotype with the severity of COVID-19, others have indicated conflicting results [15, 17, 18, 25]. Nonetheless, in our study, *ACE* gene polymorphism was not associated with disease severity, mortality, coexistence of chronic illnesses, or any medication use.

According to the results of our study, ACE levels seem not to be correlated with the gene polymorphisms, independently of the usage of ACEi/ARB drugs. In general, the ACE I/D genotype was suggested to be a genetic trait locus determining circulating ACE levels [26]. In a study analyzing patients undergoing heart or lung surgery, serum ACE concentration was found to be correlated with the ACE I/D genotype with a predominance in the DD genotype [27]. In addition, this study revealed that circulating ACE activity correlates with heart ACE but not with lung ACE in humans. However, it is known that ACE expression is regulated not only by the genetic background but also by endogenous inhibition and secretion mechanisms such as physiological factors, the biochemical environment and endothelial function [28]. Actually, it is difficult to reveal the association between the level of ACE enzyme and gene polymorphism in COVID-19 patients, as it is a hyperinflammatory state. Therefore, the clinical reflection of different polymorphisms may be altered during COVID-19.

Several studies have been conducted to clarify the association between the RAAS and COVID-19 [4–6, 29–34]. HT and DM, which are associated with unfavorable outcomes in patients with COVID-19, are closely correlated with increased activation of the ACE/Ang 2 axis. Therefore, severe forms of COVID-19 may result from a history of RAAS imbalance [35]. There are some conflicting data regarding ACE levels and activity in COVID-19 cases. While in some studies have reported a decrease [7, 36], others show that there is no significant change in ACE level or activity [9, 37, 38]. One study examining patients with COVID-19 showed that a lower level of serum ACE on admission was an independent risk factor for disease progression, while an ACE level of ≤33.5 U/L predicted a higher cumulative viral RNA and was associated with increased proinflammatory mediator levels and decreased lymphocyte counts [7]. Impaired shedding of ACE from inflamed pulmonary vascular endothelium, decreased ACE secretion from damaged endothelium due to severe inflammation and increased reactive oxygen species have an inhibitory effect on ACE activity, leading to low ACE levels. In this study, ACE levels were measured in a small group of patients and the ACE gene polymorphism was not analyzed. Similarly, another study showed that ACE levels are decreased in COVID-19 infection and the levels are correlated with interleukin 6, TNF-alpha and PAI-1 [36]. They measured ACE levels both on admission and in the follow-up of the patients, showing that ACE level was increased in the follow-up in comparison to that in active infection, but ACE2 level decreased during the resolution of COVID-19. In contrast to these studies, we found a higher level of ACE in severe disease and predicted ICU admission and mortality. To be more specific, we demonstrated that an ACE level >37.092 ng/mL predicted ICU admission with a sensitivity of 92.5%. We also found that the ACE level on admission was a predictor of mortality and ICU admission, with normalized importance ratios of 30.3% and 60.1, respectively. Although there are some theories and opinions, this is the first study to show the impact of elevated ACE levels on the worsening course of COVID-19. In support of this argument, we also showed that ACE levels were positively correlated with inflammatory markers such as ferritin (p<0.001), LDH (p<0.001), hs-CRP (p = 0.001), D-dimer (p<0.001) and procalcitonin (p = 0.002) in COVID-19 (Table 2). This is a consistent finding with an increased ACE level in severe disease. Our findings support the idea of RAAS activation in COVID-19.

The binding of SARS-CoV-2 to ACE2 may result in decreased ACE2 availability, leading to downregulation of the ACE2/Ang 1–7 axis and consequent activation of the ACE/Ang 2 axis [35, 39, 40]. In a study, Ang 2 levels were significantly increased in critically ill COVID-19 patients [41]. In addition, ACE2, which associates with AT1Rs to form a cell membrane complex, is released from the cell surface into the cell by binding to COVID-19 [35]. Thus, an Ang 2-AT1R-mediated inflammatory response occurs and induces direct parenchymal injury in lungs. It seems that RAAS imbalance might not only be a consequence of the disease but also a crucial step of COVID-19 pathogenesis. It has been proposed that ACE2 expression was upregulated with the use of RAAS inhibitors [42]. Thus, the binding and internalization of the SARS-CoV-2 virus to the cell may be facilitated. Remarkably, several studies have shown that RAAS inhibitors decrease the severity and mortality of COVID-19 by blocking the Ang 2-AT1R-mediated inflammatory response in the lungs. Mechanistically, ACEi and ARB decreases the degradation of the ACE2 protein, leading to the prevention of acute lung injury [32–34]. In a previous study analyzing ACE2 activity, the RAAS was involved in COVID-19 in hypertensive patients independent of RAAS blockade treatment [43]. Another study showed an increased ACE activity in fatally ill patients with COVID-19, which indicated dysregulation of the RAAS in the infection [8]. They also showed that none of the patients under ACEi/ARB treatment died, indicating a possible protective role of ACEi/ARB in COVID-19. However, our findings did not support the association between ACEi/ARB use and mortality or ICU admission. Moreover, ACE levels were similar in patients with or without HT (p = 0.374) and with HT using or not using ACEi/ARB (p = 0.999). Similar to our study, a large study showed that plasma ACE2 concentrations were independent of ACEi/ARB use in patients with cardiovascular disease or other risk factors [44]. Therefore, future studies including a large spectrum of hypertensive patients under ACEi/ARB treatment are needed to clarify this issue.

In addition to the effect of T2DM on the severity of COVID-19, antidiabetic agents were also investigated in the context of prognosis and mortality of the disease [21, 45–47]. The DPP4 enzyme is a major regulator of the incretin system, and hence, DPP4i has become one of the major therapeutic choices in the management of T2DM. The DPP4 enzyme, also called CD26, together with ACE2, is a binding site that internalizes MERS-CoV corona-like virus into host cells [48–50]. Soluble DPP4 serum levels were found to decrease not only in MERS-CoV infection but also in COVID-19 infection [51]. Given the association of the DPP4 enzyme and COVID-19 infection, several studies have been conducted regarding the effect of DPP4i’s on the prognosis of COVID-19 infection [45, 52–54]. It has been proposed that DPP4 inhibitors, by altering the interaction of SARS-CoV-2 with host cells and by antifibrotic and anti-inflammatory mechanisms, may change the clinical outcomes of COVID-19 infection [52, 53]. Previous studies have suggested that preexisting treatment with DPP4 inhibitors decreases the mortality associated with COVID-19 infection in T2DM [45, 54]. A large study revealed that DPP4i use was a protective factor against hospitalization, ICU admission and mortality in COVID-19 [45]. In contrast, we found that DPP4i use was not associated with disease severity, mortality, or ICU admission. Furthermore, ACE levels were similar in patients with and without T2DM (p = 0.062) and in those with and without DPP4i treatment (p = 0.427). In a meta-analysis indicating the association of DPP4i use with lower mortality in COVID-19 infection, the protective effect of DPP4i use was attenuated by the effect of ACEi/ARB or metformin use [54]. Conversely, a meta-analysis demonstrated that administration of DPP4i in patients with diabetes and COVID-19 cannot reduce inflammation and alter the severity and mortality outcomes [55]. Besides, in a cohort study of diabetic COVID-19 patients, Khunti et al. revealed that the mortality risk was higher in those prescribed DPP4 inhibitors than in those not prescribed [56]. Because of the conflicting data in the literature, there is a need for further studies examining the effects and mechanisms of DPP4i’s on mortality and prognosis.

### Strengths and limitations

We evaluated ACE gene polymorphisms and ACE levels in a considerable number of patients. We demonstrated that elevated levels of ACE are a risk factor for poor prognosis in COVID-19 and analyzed the predictive value and importance for worsening of disease. We measured ACE levels only on admission but not during hospitalization or in the follow-ups. Furthermore, we did not measure ACE2 activity or tissue levels of the ACE enzyme. Additionally, we could not examine the effect of glucose regulation on the ACE level of COVID-19 patients with diabetes, as we could not access the glucose and HbA1c data of the patients.

## Conclusion

Our findings suggested that higher ACE levels, but not *ACE* gene polymorphism, were associated with the poor prognosis of COVID-19 infection. The presence of HT and T2DM and ACEi/ARB or DPP-4i use were not associated with mortality or ICU admission. Future studies including a higher number of patients with or without T2DM or HT, under the treatment with or without RAAS inhibitors and with or without DPP4i, which analyze both ACE and ACE2 levels on admission and in the follow-up, can more clearly demonstrate the association between ACE levels in COVID-19 infection.

## Supporting information

S1 Table Demographic, clinical and laboratory findings of the patients. (DOCX)

S2 Table Raw data of the study patients. (XLSX)
