# Supplementary material for: Angiotensin-Converting Enzyme (ACE) level, but not ACE gene polymorphism, is associated with prognosis of COVID-19 infection: Implications for diabetes and hypertension
Source: PLoS One. 2023 Jul 11;18(7):e0288338. doi: 10.1371/journal.pone.0288338 (PMC10335690; doi:10.1371/journal.pone.0288338)
Supplement: S1 Table — (DOCX) [file pone.0288338.s001.docx]

**S1 Table. Demographic, clinical and laboratory findings of the patients.**

| **(n = 266)** | | **Mean (SD.)** |  | **Median (min-max)** |
| --- | --- | --- | --- | --- |
| **Age (year)** | | 57.6 (14.2) |  | 57 (20 -91) |
|  | | **n** |  | **%** |
| **Sex (Female)** | | 114 |  | 42.9% |
| **Disease severity** | |  |  |  |
|  | Mild | 33 |  | 12.4% |
|  | Moderate | 149 |  | 56.0% |
|  | Severe | 84 |  | 31.6% |
| **Exitus** | | 11 |  | 4.1% |
| **ICU admission** | | 33 |  | 12.4% |
| **CT findings** | | 236 |  | 88.7% |
| **Oxygen demand** | | 173 |  | 65.0% |
| **Duration of hospitalization (day)** | | 14.2 (12.9) |  | 11 (2-116) |
| **Respiratory rate (/min)** | | 25 (6.4) |  | 22.5 (14-50) |
| **T2DM** | | 75 |  | 28.2% |
| **HT** | | 116 |  | 43.6% |
| **CAD** | | 38 |  | 14.3% |
| **COPD/Asthma** | | 28 |  | 10.5% |
| **CVA** | | 7 |  | 2.6% |
| **DPP-4i** | | 16 |  | 6.0% |
| **ACEi/ARB** | | 74 |  | 27.8% |
| **ACE gene** | |  |  |  |
|  | DD | 87 |  | 32.7% |
|  | ID | 137 |  | 51.5% |
|  | II | 42 |  | 15.8% |
| **I allele** | | 179 |  | 67.3% |
| **D allele** | | 224 |  | 84.2% |
|  | |  |  |  |
|  |  | **Mean (SD.)** |  | **Median (min-max)** |
| **ACE level (ng/mL)** | | 21.3 (11.2) |  | 19 (0.7-50) |
| **Lymphocyte (/mm^3^)** | | 1164.7 (684.1) |  | 1000 (200-7200) |
| **Lymphocyte (%)** | | 20.4 (9.9) |  | 18.7 (3.3-51.6) |
| **Neutrophil (/mm^3^)** | | 4516.5 (2740.5) |  | 3800 (600-21300) |
| **Neutrophil (%)** | | 69.7 (11.7) |  | 71.3 (34.3-94.6) |
| **NLR** | | 5 (4.3) |  | 3.7 (0.7-31.5) |
| **Platelet (/mm^3^)** | | 200.6 (84.4) |  | 186.5 (36-562) |
| **Ferritin (mcg/L)** | | 328.7 (406.1) |  | 185.8 (2.8-2770) |
| **LDH (U/L)** | | 310.7 (148.8) |  | 269.5 (131-979) |
| **Hs-CRP (mg/dL)** | | 62.5 (68.4) |  | 35.4 (0.9-412) |
| **D-dimer (mcg/mL)** | | 1.1 (2.2) |  | 0.6 (0.2-20) |
| **Procalcitonin (ng/mL)** | | 0.3 (1.7) |  | 0.1 (0-20.5) |
| ICU, intensive care unit; CT, computed tomography; T2DM, type 2 diabetes mellitus; HT, hypertension; CAD, coronary artery disease; COPD, chronic obstructive pulmonary disease; CVA, cerebrovascular accident; DPP4i, dipeptidyl peptidase-4 inhibitor; ACEi, angiotensin converting enzyme inhibitor; ARB, angiotensin II receptor blocker; ACE, angiotensin converting enzyme; NLR, neutrophil/lymphocyte ratio; LDH, lactate dehydrogenase; hs-CRP, high sensitive C‐reactive protein.  SD., Standard deviation. | | | | |
